# Preparation and Evaluation of Gelatin-Chitosan-Nanobioglass 3D Porous Scaffold for Bone Tissue Engineering

**DOI:** 10.1155/2016/9825659

**Published:** 2016-01-14

**Authors:** Kanchan Maji, Sudip Dasgupta, Krishna Pramanik, Akalabya Bissoyi

**Affiliations:** ^1^Department of Ceramic Engineering, National Institute of Technology, Rourkela 769008, India; ^2^Department of Biotechnology and Medical Engineering, National Institute of Technology, Rourkela 769008, India

## Abstract

The aim of the present study was to prepare and characterize bioglass-natural biopolymer based composite scaffold and evaluate its bone regeneration ability. Bioactive glass nanoparticles (58S) in the size range of 20–30 nm were synthesized using sol-gel method. Porous scaffolds with varying bioglass composition from 10 to 30 wt% in chitosan, gelatin matrix were fabricated using the method of freeze drying of its slurry at 40 wt% solids loading. Samples were cross-linked with glutaraldehyde to obtain interconnected porous 3D microstructure with improved mechanical strength. The prepared scaffolds exhibited >80% porosity with a mean pore size range between 100 and 300 microns. Scaffold containing 30 wt% bioglass (GCB 30) showed a maximum compressive strength of 2.2 ± 0.1 MPa. Swelling and degradation studies showed that the scaffold had excellent properties of hydrophilicity and biodegradability. GCB 30 scaffold was shown to be noncytotoxic and supported mesenchymal stem cell attachment, proliferation, and differentiation as indicated by MTT assay and RUNX-2 expression. Higher cellular activity was observed in GCB 30 scaffold as compared to GCB 0 scaffold suggesting the fact that 58S bioglass nanoparticles addition into the scaffold promoted better cell adhesion, proliferation, and differentiation. Thus, the study showed that the developed composite scaffolds are potential candidates for regenerating damaged bone tissue.

## 1. Introduction

Designing new biomaterials that can mimic the micro/nanostructure and chemical composition of the native extracellular matrix (ECM) is one of the most attractive and important research areas in the field of tissue engineering. ECM governs cell attachment, growth, migration, and differentiation, as well as the formation of new tissues [1]. Scaffolds that mimic ECM of bone should ideally be processed from biomaterials with adequate properties such as biocompatibility, osteoconduction, bioactivity, osteoinduction, and biodegradation [2]. Three-dimensional (3D) porous structure of scaffolds provides the necessary support for cells to proliferate and maintain their differential function, and its architecture ultimately governs the geometry of a new bone [3]. Moreover, scaffolds for bone regeneration should mimic bone morphology, structure, and function. Bone is composed of calcium phosphate (69–80 wt%, mainly hydroxyapatite), collagen (17–20 wt%), and other components (water, proteins, etc.) [4]. For this reason, composites based on apatite crystals and natural biopolymers have received increasing attention in bone tissue engineering applications [5, 6].

Biocomposites based on biodegradable polymers and bioactive ceramics have been developed for applications in bone repair and reconstruction. Several polymers such as polylactic acid (PLA), polyglycolic acid (PGA), polylactic-coglycolic acid (PLGA), gelatin, alginate, and chitosan (CH) are widely used for this purpose because of their proven biocompatibility and complete bioresorbability [7–13].

Chitosan (CS) is a natural biopolymer extracted from crustacean. CS is a polysaccharide-type biological polymer possessing reactive amine and hydroxyl groups that promote osteoblast growth and* in vivo* bone formation [14–17]. Chitosan plays an important role in the attachment, differentiation, and morphogenesis of osteoblasts, the bone forming cells, because of its structural similarities with glycosaminoglycans, a major component of bone and cartilage [18–21]. Despite its tremendous promise in bone tissue engineering application, the poor mechanical properties of chitosan limits its clinical application in weight bearing bones, which has been addressed by the addition of bioceramics in chitosan scaffolds [22].

Gelatin, a product from partial hydrolysis of collagen, has gained interest in biomedical engineering, mainly because of its biocompatibility and biodegradability. Since it contains Arg-Gly-Asp (RGD)-like sequences that promote bone cell adhesion and migration, it has been blended with chitosan to improve the biological activity of composite scaffold [23, 24]. Gelatin-chitosan scaffold has been tested for the regeneration of various tissues including skin [25], cartilage [26], and bone [27].

Bioactive glasses are a group of bioactive ceramic materials with good bioresorbability. When immersed in a physiological solution, these bioactive materials can form hydroxyl carbonate apatite (HCA) layer that is chemically and compositionally similar to the mineral phase of human bone. The formation of apatite layer triggers chemical bonding between implant biomaterials and bone tissues [28–31]. Shalumon et al. [32] investigated the effect of nanoscale bioactive glass and hydroxyapatite incorporation in PCL/chitosan nanofiber for bone and periodontal tissue engineering. Gentile et al. [33] in their study found that SiO_2_-P_2_O_5_-CaO-MgO-Na_2_O-K_2_O containing bioglass in 70 wt% amount in chitosan-gelatin composite exhibit excellent bioactivity. Bioactivity and mechanical properties of composite scaffold comprising chitosan (CS) and 55S bioactive glass ceramic nanoparticles were reported by Peter et al. [34]. In another study Peter et al. [35] investigated the effect of 55S bioglass nanoparticle addition on physiochemical properties of chitosan-gelatin scaffold. The effect of 45S5 BG in Chi-Gel based scaffold on mesenchymal stem cell activity was reported elsewhere [36]. Previous reports demonstrated that 58S-BG containing 60 mol% SiO_2_– 33 mol% CaO– 7 mol% P_2_O_5_ is suitable for bone repair due to its excellent biocompatibility, bioactivity, and biodegradability [37, 38]. None of the studies was focused on investigating the effect of addition of 58S bioglass nanoparticles into chitosan-gelatin based scaffold on mechanical properties and mesenchymal stem cell activity onto the scaffold.

Here we report the effect of compositional variation amongst chitosan, gelatin, and synthesized 58S bioglass nanoparticles on physicochemical properties, microstructure, mechanical properties, and bioactivity of the prepared scaffold. Optimization of the composition of the prepared scaffold was performed evaluating the pore size and its distribution, biodegradation kinetics, and mechanical properties. In detail scaffold-MSCs* in vitro* interaction was investigated using SEM study on cell cultured specimens, MTT assay, and immunocytochemistry after optimization of its physicochemical and mechanical properties.

## 2. Materials and Method

### 2.1. Materials

Chitosan (Mw = 2.46 × 10^5^, degree of deacetylation = 85%) and gelatin were purchased from Sigma Aldrich (USA). Tetraethyl orthosilicate (TEOS: C_8_H_20_O_4_Si), triethyl phosphate (TEP: C_6_H_15_O_4_P), and calcium nitrate tetra hydrate (Ca(NO_3_)_2_·4H_2_O) were purchased from Merck (India). Glacial acetic acid (96%) and ammonia solution (NH_4_OH) were procured from LOBA chemical (India). Glutaraldehyde (GA) (C_5_H_8_O_2_) and 0.1 M nitric acid (HNO_3_) were purchased from Merck Inc. (India).

### 2.2. Sol-Gel Preparation of 58S Bioactive Glass

The composition of the studied bioactive glass was 57.44% SiO_2_, 35.42% CaO, and 7.15% P_2_O_5_ in molar percentages and composition was chosen based on the ternary phase diagram of CaO-SiO_2_-P_2_O_5_ [39, 40]. In brief, 14.8 g of tetraethoxysilane (TEOS) was added to 30 mL of 0.1 M nitric acid, and the mixture was allowed to react for 30 min for maximum completion of acid hydrolysis of TEOS. Distilled water was added to the solution and allowed to mix until the solution became clear. The H_2_O : (TEOS) molar ratio was 12 : 1. After 30 minutes, 0.85 gm of triethyl phosphate (TEP) was added to the stirring solution followed by the addition of 7.75 gm of calcium nitrate after another 20 minutes. The solution was then stirred for an hour. The mixture was cast in a cylindrical Teflon container and kept sealed for a week at room temperature to allow hydrolysis and a polycondensation reaction to occur in course of gelation of the sol. The gel was dried at 120°C for two days in an oven. The dried powder was heated for 24 h at 700°C for nitrate elimination and stabilization of gel. Subsequently, the powders were ball milled by planetary milling (Fritsch Company, Germany) at 400 rpm for six h.

### 2.3. Preparation of Porous Gelatin-Chitosan/Bioglass (GCB) Composite Scaffold

The GCB scaffolds were prepared with varying amount of synthesized 58S-BG nanopowder keeping the amount of gelatin constant at 30 wt% as listed in Table 2. Our previous work [41] indicated that increase in nanoceramic phase content beyond 30 wt% in gelatin-chitosan matrix resulted in <50 *μ*m pore size in the prepared scaffold and rendered it not ideal for cell ingrowth, hence exhibiting osteoinduction. Similarly, scaffold's average pore size decreased below 40 *μ*m on increasing gelatin content higher than 30 wt% with nanoceramic phase content varying between 10 and 30 wt% in the scaffold. As we found that 30 wt% gelatin content scaffold exhibited the highest compressive strength, our objective in this study is to evaluate the physicochemical and mechanical properties of the scaffold on varying bioglass content from 10 wt% to 30 wt% keeping gelatin content fixed at 30 wt%.

**Figure 1:**
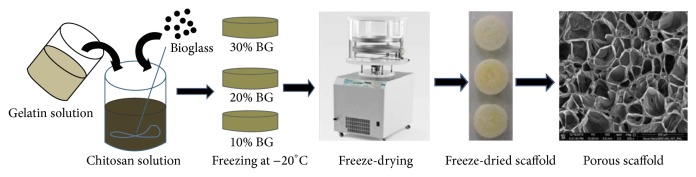
Fabrication procedure for the GCB scaffolds.

Figure 1 illustrates the preparation processes of GCB scaffolds. First, chitosan (CS) solution was prepared by dissolving required amount of medium molecular weight chitosan in a solution containing 4 mL of acetic acid and 96 mL of deionized water with stirring for 5 h to get a perfectly transparent solution, as we have prepared in our previous work [42]. Separately, glutaraldehyde (GA) solution was prepared by dissolving 0.5 mL (50%) glutaraldehyde in 100 mL deionized water. Next required amount of gelatin was dissolved in deionized water with continuous stirring at 40°C for four h. This solution was then added to the 2 wt% chitosan solution. Different percentage (10%, 20%, and 30%) of 58S-BG was then added into the gelatin-chitosan solution and stirred for 4 h. The resulting slurry (gelatin-chitosan-bioglass) was put in PTFE cylindrical mould and then rapidly prefreezed at −20°C to solidify the water and kept overnight. Next, the frozen samples were lyophilized using a freeze-dryer (Labconco, USA) at −52°C for 24 h. Finally, samples were soaked in 1 wt% GA solution for 24 h and then carefully washed with deionized water to remove the remaining amount of GA. The cross-linked scaffold was further treated with sodium borohydride aqueous solution to block residual aldehyde groups and then washed with ethanol-NaOH followed by deionized water. The washed porous scaffold was again freeze-dried to obtain the hybrid network of gelatin-chitosan-bioglass.

### 2.4. Characterization

#### 2.4.1. Particle Size Analysis Using Dynamic Light Scattering (DLS)

Particle size of synthesized bioglass nanopowder was measured using (DLS) technique by Malvern Zetasizer (ZEN3690, USA). Two mg of dried powder was suspended in 50 mL water after ultrasonication, and 2 mL of the suspension was taken in a cuvette and used for the measurement of particle size.

#### 2.4.2. X-Ray Diffraction (XRD) Analysis

The phases of composite scaffolds were characterized by X-ray diffraction (XRD) using fully automated X-ray diffractometer (Panalytical, USA) fitted with Ni filter. The diffraction patterns were recorded with a XRD analyser using Cu-K*α* radiation (*λ* = 1.542 Å) at 35 kV and 40 mA. The samples were scanned in the interval of 10° < 2*θ* < 70° at a scan speed of 2°/min with step size .02° in a continuous mode.

#### 2.4.3. Fourier Transform Infrared Spectroscopy (FTIR) Analysis

The characteristic peaks of the composite scaffold were analysed using Fourier transform infrared (FTIR) (Perkin Elmer, USA) spectroscopy. The scaffolds were put in a vacuum oven at 50°C for 48 h before they were ground to a suitable size for IR analysis with spectrometer. The pellet for the FTIR measurement was prepared by mixing the sample (2 mg) with 200 mg of IR-grade KBr. The absorption spectra were measured using IR spectrometer at a wavenumber of 4000–400 cm^−1^ with a resolution of 1 cm^−1^.

#### 2.4.4. Scanning Electron Microscope (SEM) Analysis

The composition of prepared bioglass powder was studied using energy dispersion X-ray (EDX) analysis. The morphology and microstructure of the scaffolds have been observed using field emission scanning electron microscopy (FESEM) (Novanano 450, FEI, Netherlands) operated at 78 *μ*A, 15 KV. The surface of the scaffolds was coated with thin layer of gold (Au) and then placed inside the FESEM chamber.

#### 2.4.5. Mechanical Behaviour (Compression Test)

The mechanical properties of the composite scaffolds were determined using a Universal Testing Machine (Tinius Olsen, UK) with a crosshead velocity of 1 mm min^−1^ and a 1000 N load cell. For compressive testing, the samples (*n* = 5) were cylinders of approximately 15 mm in diameter and 6 mm in height in accordance with the compression mechanical test guidelines set in American Standard Test and Measurement (ASTM F 451-95). Specimens were compressed to ~40% of their original thickness and the values were expressed as the means ± standard error.

#### 2.4.6. Porosity of Scaffold

The porosity of composite scaffold (1 × 1 × 1 cm^3^) was measured using Archimedes principle with xylene as liquid medium using the following equation:

(1) $$\begin{array}{c} \text{Porosity }\left(\;\%\;\right)=\frac{\text{Soaked weight of the sample}-\text{Dry weight of the sample}}{\text{Soaked weight of the sample}-\text{Suspended Weight of the sample in liquid}}\times 100\%, \end{array}$$

where *W*_1_ is the weight of the sample in air, *W*_2_ is the soaked weight of the sample in xylene, and *W*_3_ is the weight of the sample suspended in xylene. Pore size distribution in the scaffold was measured using Hg porosimeter (Quanta Chrome, USA).

#### 2.4.7. Swelling Index

The swelling properties of the chitosan-gelatin scaffolds were investigated according to a method described earlier [43]. Briefly, the scaffolds were fully immersed in the phosphate buffer solution (pH 7.4) at room temperature for 60 min. Samples were recovered after every 10 minutes of soaking in PBS and excess water onto scaffold surface was extracted out using distilled paper. The wet weight of the scaffold (*W*_*s*_) was determined using an electronic balance and recorded. Next the swollen scaffold was dried in an oven at 50°C overnight and weighed and the dry weight was recorded as *W*_*d*_. Three scaffolds were selected for each period and weighed under the same conditions. The swelling percentage of scaffolds was calculated as follows:

(2) $$\begin{array}{c} S=\left[\;\frac{\left(W_{s}-W_{d}\right)}{W_{d}}\;\right]\times 100\%. \end{array}$$

#### 2.4.8. *In Vitro* Biodegradability

The scaffolds were immersed into the phosphate buffer fluid PBS solutions for degradation assessment by monitoring the weight loss. The scaffolds were immersed in PBS (pH 7.4) at 37°C. At a predetermined day interval up to 14 days, after being incubated for various time durations the scaffolds were taken out from the medium, washed with distilled water, and freeze-dried. The degradability ratio *D* was calculated as follows:

(3) $$\begin{array}{c} D=\left[\;\frac{\left(W_{o}-W_{t}\right)}{W_{o}}\;\right]\times 100\%, \end{array}$$

where *W*_o_ denotes the original weight and *W*_*t*_ is the weight of the sample after immersing in PBS up to day *t*. Each biodegradation experiment was conducted on three samples, and the average value was taken as the percentage of biodegradation.

### 2.5. Cell Culture Study

#### 2.5.1. Attachment and Morphology of Cell on GCB Composite Scaffolds

Human umbilical cord mesenchymal stem cells (MSCs) were grown in a Dulbecco's modified Eagle's medium (DMEM, Sigma Aldrich, UK) supplemented with 10% fetal bovine serum (FBS, Sigma Aldrich, UK) and 100 U/mL penicillin-streptomycin. Cells were propagated in a 75-cm^2^ T-flask (Corning, NY, USA) in a humidified atmosphere containing 5% CO_2_ at 37°C. Ethylene oxide sterilized GCB 30 scaffold disks were placed inside a 24-well plate and MSCs were seeded at 1 × 10^6^ cells per well in the scaffolds. Cell culture medium was replaced after every 3 days. At 3, 5, and 7 days after cell seeding, GCB 30 disks were taken out from the cell culture medium. GCB 30 disks were first briefly rinsed in cold 0.1 M phosphate buffer solution (pH 7.4) twice. The cells were then fixed in 2.5% glutaraldehyde in 0.1 M phosphate buffer for 3 h. The fixative was removed, and cells were rinsed in 0.1 M phosphate buffer and in distilled water. Afterwards, the MSC cultured GCB 30 disks were dehydrated in a graded series of ethanol solution (50%, 70%, 80%, and 90% absolute ethanol), 100% pure acetone, and hexamethyldisilazane (HMDS). Next the samples were desiccated under vacuum and were coated with gold before the cell attachment; spreading and morphology were investigated using FESEM at 10 KV.

#### 2.5.2. Analysis of Viability Differentiation of MSc Cultured onto the Scaffolds

Cell viability of MSCs cultured onto the scaffolds was determined after performing [3-(4,5-dimethylthiazol-2-yl)-2,5-diphenyltetrazolium bromide] (MTT) assay. We note that, for the MTT assay, the amount of produced formazan crystal is proportional to the number of viable cells present in the scaffold [44]. In brief, the scaffolds were submerged in DMEM cell culture media and MSCs were seeded on each scaffold at 1 × 10^6^ cells per well and incubated for 3, 5, and 7 days with replacement of DMEM after every 3 days of culture. After each time interval of cell culture, 20 *μ*L (0.5 mg/mL) MTT solution was added onto each scaffold placed in 24-well plate. The scaffolds containing MSCs and MTT solution were then incubated for another 3.5 h at 37°C. The precipitated purple coloured formazan crystals were dissolved in 150 *μ*L of MTT solvent by shaking the plate for 15 min. Then the solutions were taken out in ependrofs and centrifuged and the supernatant was transferred to another 96-well plate to record the absorbance at 595 nm using a microplate reader (Bio-Tek ELx800).

#### 2.5.3. Inmunofluorescent Imaging of Cell Markers: Osteocalcin and RUN-X2

The expression of RunX2, an osteogenic marker, was examined using immunocytochemistry. MSCs were seeded on scaffolds and incubated as mentioned earlier. Cells on scaffolds were fixed and permeabilized using 100% methanol (precooled to −20°C) for 10 min. Blocking was done using FBS. Staining of RUN-X2 was achieved using rabbit RUN-X2 antibody (1 : 200, Abcam) and using Alexa 647 anti-rabbit (1 : 400, Invitrogen) as secondary antibody. Staining for osteocalcin was performed using rabbit anti-mouse osteocalcin (1 : 100, Abcam) and Alexa 488 anti-mouse (1 : 400, Invitrogen) as a secondary antibody. Counter staining with diammonium propidium iodide (DAPI) was performed for 2 min after washing with PBS. To trace actin filament in the cell, green phalloidin Alexa Fluor 488 counter stained with DAPI was used. Finally the samples were mounted with Fluorsave Vectashield mounting medium with DAPI. Samples were mounted on glass slides and confocal images of the samples were acquired using Leica TCS SP5 X Supercontinuum Confocal Microscope.

#### 2.5.4. Statistical Studies

All data are expressed as mean ± standard deviation. The data were compared using Student's *t*-test and differences were considered significant when ^*∗*^ *p* < 0.05. A *p* value more than 0.05 (*p* > 0.05) was taken, indicating no significant difference.

## 3. Result and Discussion

### 3.1. XRD and FTIR Analysis

**Figure 2:**
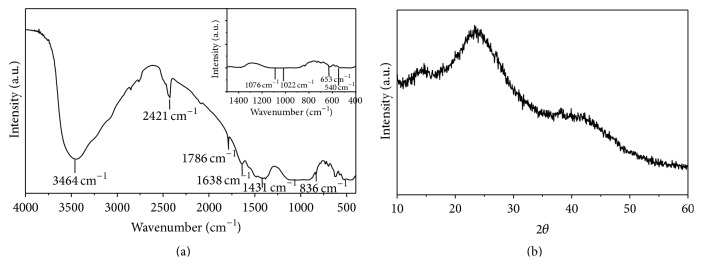
FTIR and XRD pattern of 58S bioglass powder prepared via sol-gel process.

Figure 2(a) shows the FT-IR spectrum of synthesized bioglass powder. The characteristic bands for different functional groups present in the powder are shown in Table 1. The band at 1638 cm^−1^ corresponds to carbonate (CO_3_^2−^) coming from atmospheric CO_2_ and attached to Ca^2+^. The band at 1022 cm^−1^ arises from *ν* ^3^ PO_4_, whereas the band at 653 cm^−1^ suggests the presence of *ν* ^4^ PO_4_. The band at 836 cm^−1^ was ascribed to the stretching vibration of Si–O– groups in bioglass. The absorption bands observed at 1076 cm^−1^ and 540 cm^−1^ were assigned to stretching vibration of Si–O–Si, respectively, and bending of Si–O–Si [45]. Typical absorption band at 1786 cm^−1^, 1431 cm^−1^ is attributed to the deformation mode of adsorbed molecular water in the pores. Phase analysis of the composite nanopowder was performed using XRD.  Figure 2(b) clearly indicates the amorphous nature of the synthesized bioglass powder.

### 3.2. Particle Size, Morphology, and Composition of Nanobioglass Powder

**Figure 3:**
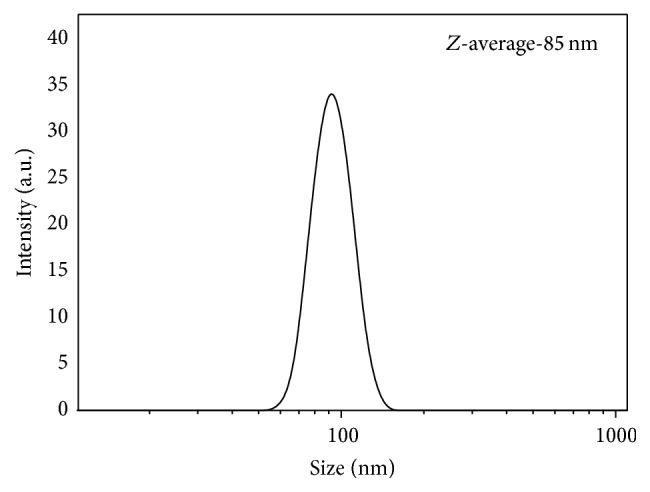
The particle size distribution of NBG measured by DLS.

**Figure 4:**
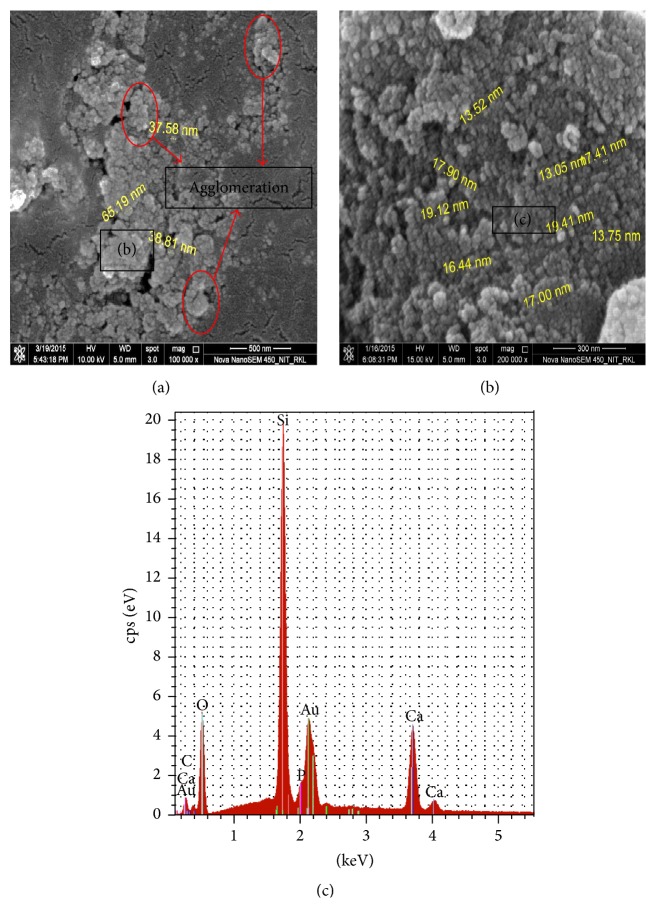
(a) FESSEM micrograph of synthesized bioglass nanopowders, (b) magnified image of particle agglomerate, and (c) elemental composition analysis of the synthesized bioglass nanoparticles.

Figure 3 represents DLS particle size measurement data of synthesized 58S bioglass nanopowder. An average particle size of 85 nm as obtained from the DLS measurement was little bit higher as compared to the particle size shown in the FESEM image in Figure 4(a). Particle size measurement by DLS technique gives the hydrodynamic diameter of the particle and also bioglass particles were to some extent in agglomerated state in the suspension that suggest higher average particle size value in the DLS measurement. From FESEM micrograph in Figure 4(b) it is evident that the bioglass particle was spherical in morphology and was agglomerated in course of drying on a carbon film (Figure 4(a)). EDS analysis in Figure 4(c) and Table 3 suggests that the synthesized powder with a composition of Si : Ca : P = 36 : 21 : 7 closely resembled the theoretical composition.

### 3.3. XRD Analysis of Composite Scaffold

**Figure 5:**
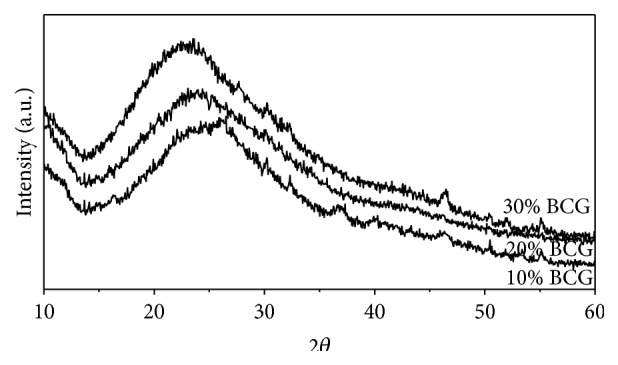
XRD analysis of composite scaffold with varying amount of bioglass powder.

Phase analysis of the composite scaffolds shown in Figure 5 was performed using XRD. The characteristic diffraction peaks for both chitosan and gelatin were suppressed by the huge amorphous peak of bioglass observed in the range between 2*θ* equal to 20°–40°. The broad amorphous peaks of bioglass confirmed that the synthesized scaffolds were predominantly amorphous.

### 3.4. Chemical Structure Study of Composite Scaffold

**Figure 6:**
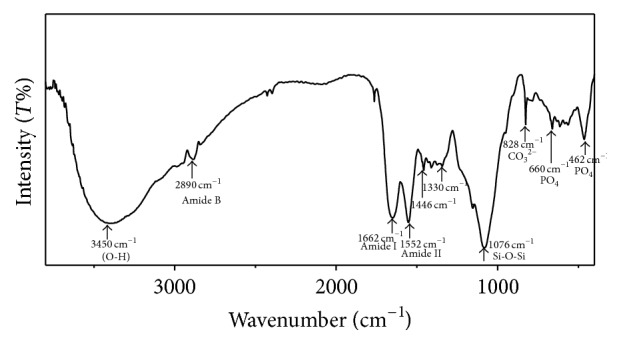
FTIR spectra of gelatin/chitosan/bioglass (GCB 10) composite scaffold.

The chemical structure of composite scaffolds was investigated by FTIR, in order to examine the chemical interactions between the gelatin, chitosan, and bioglass phases, as shown in Figure 6. Apart from corresponding peaks of PO_4_^3−^ at 462 and 660 cm^−1^, Si–O–Si in bioglass at 1076 cm^−1^ and CO_3_^2−^ bands at 828 cm^−1^, bands for amide I at 1662 cm^−1^, amide III at 1232 cm^−1^, and carboxylate at 1446 cm^−1^ in gelatin, a distinct band at 1552 cm^−1^ was detected. This absorption band signifies the formation of –C=N– bond due to intergelatin and chitosan-gelatin cross-linking to develop 3D interconnected network in the scaffold [46]. The appearance of an amide I mode at 1662 cm^−1^ indicates that bioglass-gelatin composites adopted a predominantly *α*-helical protein configuration, and this was further confirmed by the appearance of amide II mode at 1552 cm^−1^. The band at 1330 cm^−1^ is attributed to the chemical bond formation between carboxylate group in gelatin and Ca^+2^ ion in BG to bind the particulate reinforced composite scaffold together [47, 48]. These interactions between the gelatin, chitosan, and silica phases would enhance the stability of the composite gelatin-chitosan-bioglass hybrid scaffolds in a water-based environment.

### 3.5. Microstructure Study

**Figure 7:**
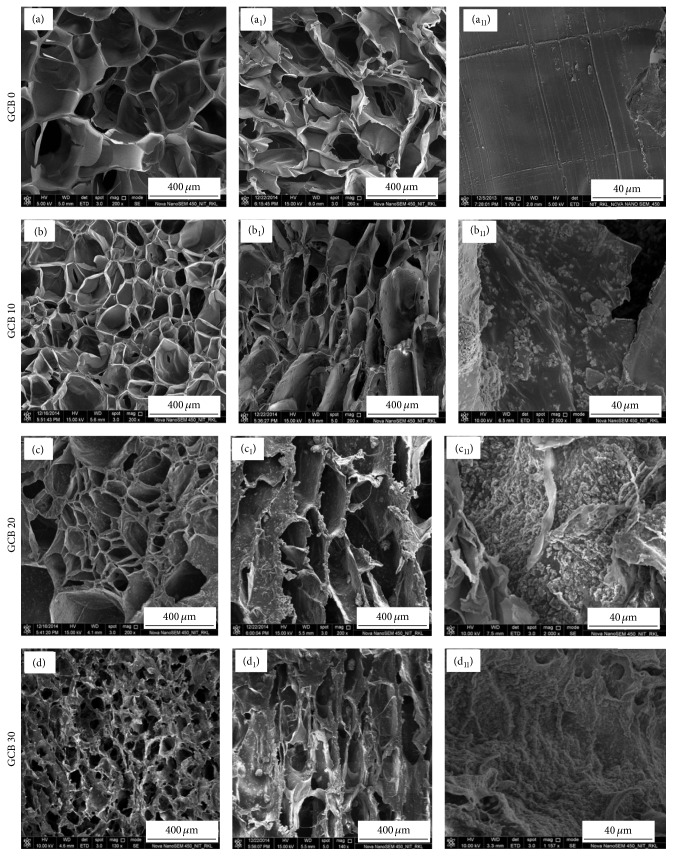
FESEM microstructure of composite scaffolds. Cross-sectional Figures 7((a)–(d)), longitudinal Figures 7((a_1_)–(d_1_)), and pore wall distribution Figures 7((a_11_)–(d_11_)).

The physical characteristic of a scaffold can be described by its average pore size, pore interconnectivity, and pore shape. Pores are necessary for the bone tissue formation because they allow migration and proliferation of osteoblast and mesenchymal stem cells, as well as the proper vascularization of the implant [49]. The morphology of BCG scaffolds before and after n-bioglass incorporation is shown in Figure 7. FESEM micrograph of composite scaffolds (Figure 7) showed that scaffolds were macroporous in nature. The porosity was found to vary from 81 to 89% depending on the percentage addition of bioglass powder. The pore size of GCB scaffolds with 10 wt% bioglass was around 200–250 *μ*m. As the bioglass content increased to 30 wt%, the pore size was reduced to 100–120 *μ*m and pore shape became irregular. With the augmentation of bioglass content in GCB slurry, the interactions between bioglass particles and gelatin increased with consequent increase in solution viscosity. Thus as we go from 10 to 30 wt% bioglass content in scaffold, a higher and higher force was necessary for GCB slurry to be expelled by water molecules, so the ice crystals growth was hindered during freezing of slurry resulting in reduced size of intertrapped ice blocks and scaffolds with smaller pores were formed during subsequent sublimation [50]. Top views (Figures 7(a)–7(d)) and side views (Figures 7(a_1_)–7(d_1_)) representations of the scaffold are shown in Figure 7 that were used to calculate the average pore size. In particular, with an increase in bioglass content, increasing amount of bioglass particles was deposited onto the chitosan-gelatin walls (Figures 7(a_11_)–7(d_11_)) as confirmed subsequently by FESEM examination. All results demonstrate that the prepared scaffolds exhibited a pore size distribution in the range of 100–250 microns possessing rough pore wall, crucial for protein and cell adhesion.

### 3.6. Analysis of Pore size and Its Distribution in the Scaffold

**Figure 8:**
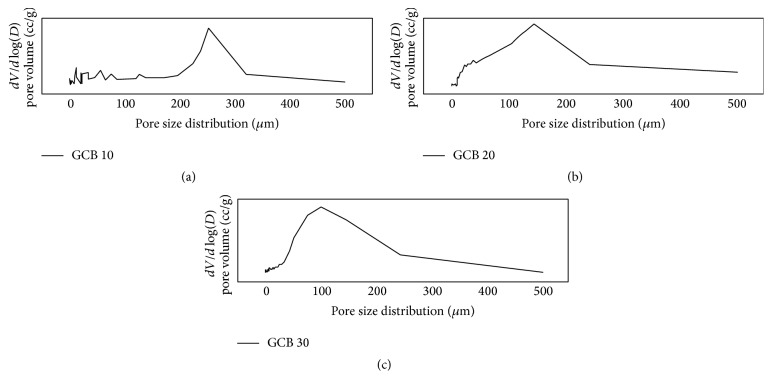
Pore size distribution data of gelatin-chitosan-bioglass scaffold: (a) GCB 10, (b) GCB 20, and (c) GCB 30.

Table 4 indicates the total porosity and means pore diameter values of the different scaffold with varying composition. The mean porosity value which varied between 81% and 89% suggests that compositional variation did not affect the total porosity in a significant manner (all *p* values > 0.05). Pore size distribution data in Figure 8 also shows the unimodal pore size distribution for all the prepared scaffold. The most frequent pore diameter in the scaffold was decreased from 253 *μ*m to nearly 100 *μ*m as we increase bioglass content from 10% to 30% in GCB slurry. All the scaffold showed the ideal pore size distribution for exhibiting osteoinductivity and osteoblast or stem cell ingrowth into the scaffold as the pore size distribution in the scaffold falls within 100–400 *μ*m.

### 3.7. Mechanical Properties

**Figure 9:**
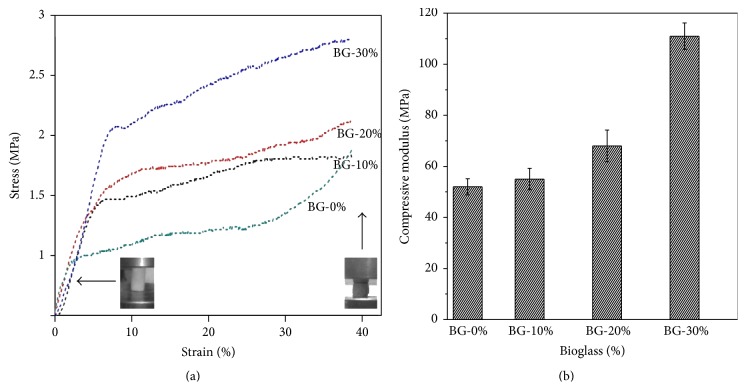
Mechanical properties of GCB scaffolds prepared at different bioglass concentrations and acquired in (a) compression test and (b) Young's modulus. The corresponding porosity and moduli are shown in Table 5.

Figure 9(a) describes the stress-strain behaviour of GCB composite scaffolds under compression. The stress-strain curve can be decomposed in two stages. The first is the elastic region before yield point and second is the postyield stage, that is, deformation region and then densifying region where the pore wall collapses. In this case, compressive strength of the scaffold is denoted by the onset of deformation region in load elongation curve. With increase of bioglass concentration from 10% to 30%, both compressive strength and elastic modulus continued to enhance significantly as in Table 5. The compressive strength and elastic modulus of pure gelatin scaffold are only 0.8 ± 0.16 MPa and 50 ± 5.23 MPa, respectively (Table 5). After incorporating 30 wt% bioactive glass particles in gelatin-chitosan matrix, the compressive strength and modulus, respectively, increased to 2.2 ± 0.02 MPa and 111 ± 12.09 MPa, respectively. The composite scaffold was elastic to yield point but after yield point some microcrack occurred. The interaction between bioglass particles and polymer network bridges the cracks in the scaffold which result in postponed final fracture. It is noteworthy that the slope of the deformation region in 30 wt% bioglass loaded scaffold was the maximum that was due to enhanced resistance from the ceramic particle to crack propagation.

### 3.8. Biodegradation Study

**Figure 10:**
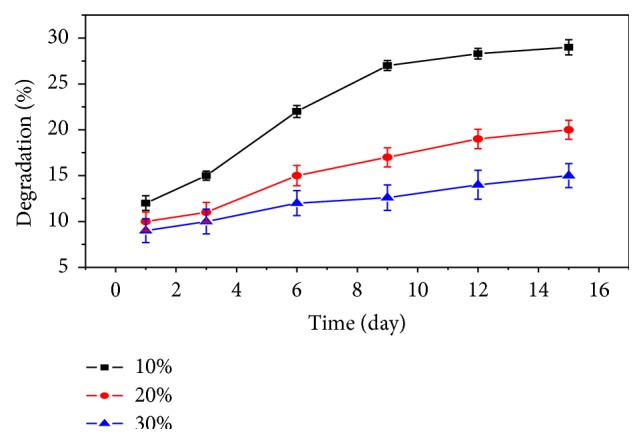
*In vitro* degradation of the GCB with different bioglass contents in PBS.

Scaffold materials are expected to be biodegradable and bioresorbable with a proper rate to match the speed of new bone tissues formation. The controlled and steady degradation behaviour of bone scaffold in physiological environments plays an important role in the regeneration of new bone tissues. The* in vitro* degradation of GCB scaffold in phosphate buffer solution (PBS) at 37°C is an important study to assess its resorbability under physiologically relevant conditions. For long-term mechanical stability and reliability, scaffold material should exhibit slow and controlled degradation behaviour.  Figure 10 demonstrates the effect of bioglass contents on the degradation rate of the scaffold. With an increase in the bioglass content, the degradation rate in PBS solution at 37°C decreased significantly. The bioglass particulate interacted through hydrogen bonded and ionic interaction with the water molecules and weakened the interaction between the gelatin macromolecule and water. The presence of bioglass also acted as a physical crosslinking sites to enhance the stability of the chitosan-gelatin network. Thus, the degradation rate and as a result strength retention in the physiological environment may be controlled by adjusting the bioglass contents in the scaffold.

### 3.9. Swelling Studies

The ability of the scaffold to swell plays an important role during the* in vitro* cell culture studies. Swelling of scaffold allows absorption of body fluid and transfer of cell nutrients and metabolites inside the scaffold. Swelling also increases the pore size and total porosity, thus maximizing the internal surface area of the scaffolds for cell infusion and attachment. However, swelling under physiological condition must be controlled; otherwise it may cause weakening and rapid degradation of the bone scaffold.

**Figure 11:**
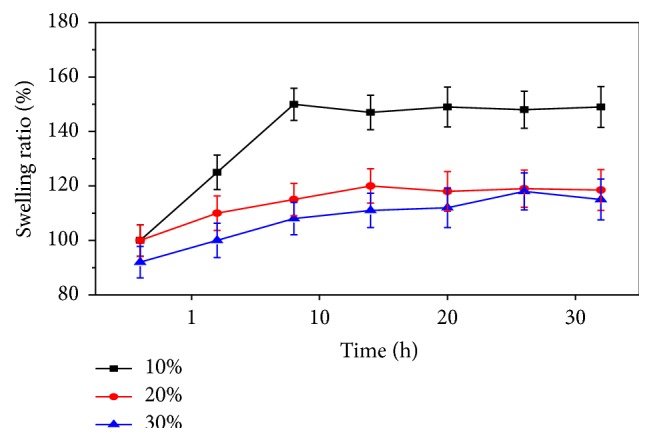
Effect of bioglass on swelling behaviour of GCB composite scaffolds as a function of time.

Figure 11 shows that swelling in the GCB scaffold increased initially and gradually attained saturation after 10 hours of soaking in PBS at 37°C. The degree of swelling was found to decrease with increasing bioglass amount in the scaffold that may be attributed to the stronger bonding interactions between cationic sites of inorganic phase and carboxylate group of gelatin in the polymeric imparting higher elasticity to the composite scaffold. Thus the increase in bioglass and decrease in hydrophilic chitosan content in the scaffold resulted in a decreased water sorption. Moreover, higher elasticity in composite scaffolds due to enhanced polymer-bioactive glass interactions resulted in slower relaxation of polymer chains that also accounted for decreasing the swelling ratio.

### 3.10. Cell Attachment Study on Scaffolds

**Figure 12:**
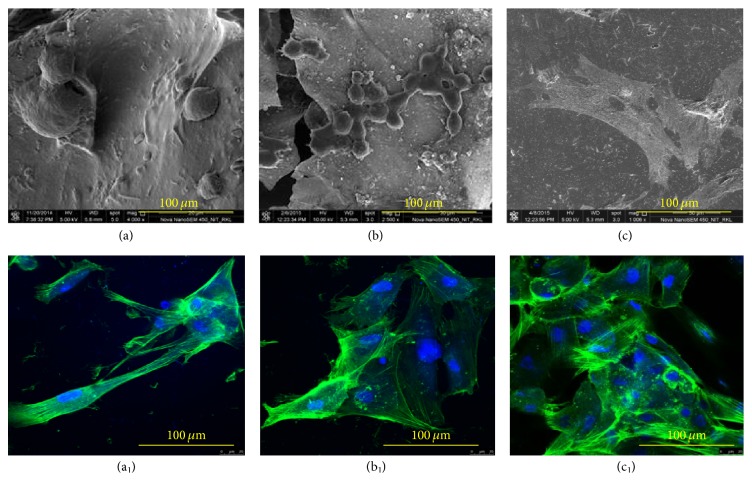
FESEM image of MSC on gelatin-chitosan-bioglass (GCB 30) for (a) 1 d, (b) 3 d, and (c) 14 d of cell culture. Confocal images of cytoskeleton of MSC on GCB 30 ((a_1_), (b_1_), and (c_1_)).

Figure 12 shows cell density and morphology after culturing MSCs on GCB 30 for different days of culture time. Cell densities, as well as number of lamellipodia and filopodia extensions from the cytoskeleton of MSCs, were increased with progress in cell culture time on the composite scaffold. The cell presented a round shape initially (Figure 12(a)) and became elongated with increasing time of culture. After 14 days, MSCs cells adopted a polygonal morphology and spread well on the scaffolds (Figure 12(c)). These results clearly exhibit that MSCs were attached, proliferated, and spread with increasing cell culture time.

Figures 12(a_1_)–12(c_1_) show confocal micrographs of MSc cells on the GCB 30 scaffolds after 1, 3, and 14 days of culture. Confocal images revealed that a higher number of cells were attached to the scaffold on increasing days of cell culture time. Figures 12(b_1_) and 12(c_1_) show a uniform interconnecting cytoskeleton of MSCs network on the GCB 30 scaffolds after culturing cells for 3 and 14 days. The increasing numbers of lamellipodia and filopodia extensions from the cytoskeleton of MSCs were evident with progress in cell culture time as in Figures 12(a_1_)–12(c_1_). Extensive networks of polymerized *β*-tubulin and F-actin filaments as well as multiple cell-cell contacts indicate a higher degree of active cell spreading, movement, and signalling events with progress in cell culture. All the results revealed higher proliferation of MSCs on GCB 30 scaffolds after 14 days of cell culture.

### 3.11. MTT Assay Study

**Figure 13:**
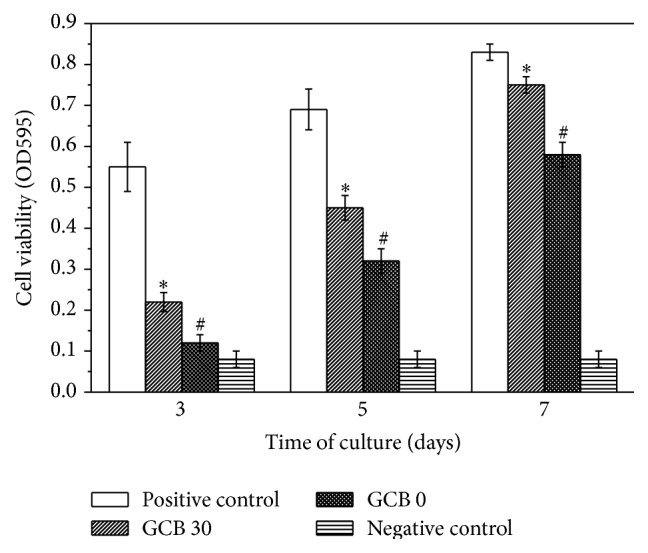
Cell viability after 3, 5, and 7 days of culture on composite scaffold of GCB 30 at 30/40/30 weight ratio as determined by MTT assay compared with glass substrate (positive control; paired with each material) on days 3 and 7. The control values were normalized to 100%. ^*∗*^ *p* < 0.05, ^#^ *p* < 0.05 compared with the corresponding control group (mean ± SE).

After culturing it on the scaffold, MSCs viability study for 3–7 days was performed using MTT assay with cell culture media as negative control and cultured MSCs on sole tissue culture plate as a positive control. Keeping up with the same trend as exhibited by the positive control the scaffold material showed higher and higher number of the viable cell with progress in cell culture time as in Figure 13. The cell density on five-day cell cultured GCB 30 sample was significantly higher (*p* value = 0.018) from that on three-day sample. Again seven-day cell cultured GCB 30 sample showed significantly higher (*p* < 0.001) viable cell density as compared to that on day 5 sample. For all incubation periods, GCB 30 presented significantly higher (*p* = 0.026) cell viability than that on GCB 0 scaffold which suggests that addition of 58S bioglass nanoparticles in the scaffold promoted better cell adhesion and proliferation. 58S nanoparticles helped in apatite mineralization onto the scaffold in the presence of cell culture media and acted as sites for cell adhesion through integrin mediated interactions from the MSCs. This showed that the selected scaffold was conducive to cell attachment and proliferation.

### 3.12. RUNX2 and Osteocalcin Expression

**Figure 14:**
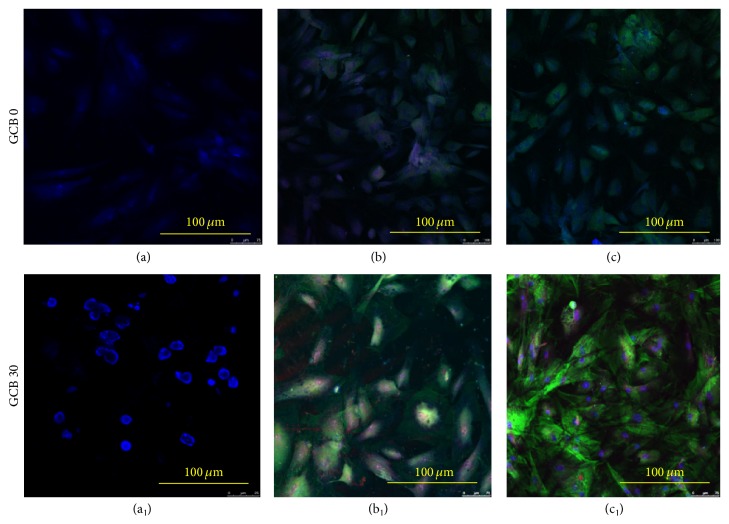
Fluorescence image of MSC's cells cultured on GCB 30 scaffolds prepared from solutions with bioglass concentration of 30% on (a) 1 day, (b) 7 days, and (c) 14 days. In fluorescence images, osteocalcin is stained green, RUNX2 is stained red, and nuclei are stained blue.

In order to assess the effect of material composition on cell differentiation and osteogenesis, expression of osteoblastic transcription factor RUN-X2 and bone noncollagenous protein osteocalcin (OC) were studied using immunofluorescent markers. As can be seen in Figures 14(a)-14(a_1_) no RUNX2 marker was visible from 1-day cell cultured sample indicating nascent and premature stage of osteoprecursor cells. RUNX2 expression appeared strongly positive in the GCB 30 scaffold cultured for 7 days, supporting the differentiation of MSCs into preosteoblast and osteoblast. There was severalfold decrease in the level of expression of most of the genes on GCB 0 scaffold (Figures 14(b)-14(c)) as compared to the GCB 30 (Figures 14(b_1_)-14(c_1_)) at different stages of differentiation, which supports the fact that bioglass addition actually increased the bioactivity of the overall scaffold. RUNX2 is a marker for osteoblast differentiation, and an increase in the specific activity of RUNX2 with progress in cell culture time in a population of mesenchymal stem cell indicates a corresponding shift to a more differentiated state (Figures 14(b_1_)-14(c_1_)).

Osteocalcin is a primary noncollagenous protein produced by osteoblasts, which signals termination of osteogenic differentiation and is commonly used to measure bone cell lineage and new bone formation [51]. Greater osteocalcin (green) deposits were seen in scaffolds of 14-day cell culture indicating the presence of higher amount of osteoprogenitor cells with progress in cell culture time (Figure 14(c_1_)). Thus, our result suggests that MSCs in GCB30 scaffold were well committed to osteogenic lineage.

## 4. Conclusions

In summary, we have successfully fabricated GCB nanocomposite scaffolds using freeze-drying method. The scaffolds were highly porous with total porosity of about 80% and average pore size in the scaffold fell to nearly 100 *μ*m from 250 *μ*m with increase in bioglass content from 10 wt% to 30 wt% in the gelatin-chitosan matrix. The bioglass particles (BG) were well distributed in gelatin-chitosan matrix, significantly improving the compressive strength and elastic modulus of the scaffolds. Thus, GCB 30 scaffold showed a compressive strength and elastic modulus value comparative to that of natural cancellous bone. It was found that the swelling behaviour of the scaffolds was reduced on the increase in 58S-BG nanopowder content in the scaffold. Biodegradation test in PBS showed that the increase in 58S-BG content resisted the biodegradability of the scaffold. Preliminary results on cell culture using MSCs suggested that cells could adhere, spread, proliferate, and differentiate very well onto GCB 30 scaffolds. MSCs were also found to transform into the new bone within 14 days of cell culture on the GCB 30 scaffold making them promising artificial bone grafts.

## Figures and Tables

**Table 1:** Component of prepared scaffolds.

Specimen name	Gel concentration (w/w) %	Chitosan concentration (w/w %)	CS-Gel/BG ratio (w/w) %	Solid loading (%)
GCB-0	30	70	100/0	40
GCB-10	30	60	90/10	40
GCB-20	30	50	80/20	40
GCB-30	30	40	70/30	40

**Table 2:** Peaks of infrared spectra assigned to synthesized bioglass powder.

Bond	Infrared frequency (cm^−1^)
Si–O–Si bending	540, 1076 cm^−1^
P–O bending vibration	1022, 653 cm^−1^
–OH	3456 cm^−1^
CO_3_^2−^	1638 cm^−1^
Adsorbed molecular water	1786, 1431 cm^−1^

**Table 3:** EDS analysis of bioglass powder.

Elements	Bioglass atomic percent (%)
Si	36.14
Ca	20.88
P	7.02
O	35.89

**Table 4:** Pore diameter and porosity of composite scaffold.

Sample name	Average pore diameter (*μ*m)	Porosity
BCG 0	400 ± 13.6	89.3 ± 7.8
BCG 10	250 ± 26.3	82.4 ± 5.0
BCG 20	160 ± 17.8	80.8 ± 3.3
BCG 30	100 ± 25.9	81.3 ± 6.1

**Table 5:** Summery of mechanical properties of GCB scaffolds prepared from solutions of different bioglass concentrations (wt%). Data refer to mean value ± standard deviation.

Bioglass scaffolds specimens	Porosity (%)	Mechanical properties	
		Elastic modulus^*∗*^ (MPa)	Compressive strength^*∗*^ (MPa) (at 40% strain)
BG 0%	89.3 ± 7.8	50 ± 5.23	0.8 ± 0.16
BG 10%	82.4 ± 5.0	55 ± 7.12	1.2 ± 0.01
BG 20%	80.8 ± 3.3	68 ± 10.10	1.6 ± 0.01
BG 30%	81.3 ± 6.1	111 ± 12.09	2.2 ± 0.02

^*∗*^Except between sample BG 0% and BG 10% (*p* > 0.05), all values in each mechanical property category were found to be significantly different from each other (*p* < 0.05, by Student's *t*-test, *n* = 5).
